# Synthesis of α-Diimine Complex Enabling Rapidly Covalent Attachment to Silica Supports and Application of Homo-/Heterogeneous Catalysts in Ethylene Polymerization

**DOI:** 10.3390/ijms241713645

**Published:** 2023-09-04

**Authors:** Xiaobei Zhao, Yanhui Hou, Linlin Ye, Kening Zong, Qingming An, Binyuan Liu, Min Yang

**Affiliations:** 1 Hebei Key Laboratory of Functional Polymers, Institute of Polymer Science and Engineering, Hebei University of Technology, Tianjin 300130, China; 2 State Key Laboratory of Separation Membranes and Membrane Processes, School of Material Science and Engineering, Tiangong University, Tianjin 300160, China

**Keywords:** α-diimine nickel(II) catalyst, heterogeneous catalysis, covalent attachment, branched polyethylene, silica support

## Abstract

For covalent attachment-supported α-diimine catalysts, on the basis of ensuring the thermal stability and activity of the catalysts, the important problem is that the active group on the catalyst can quickly react with the support, anchoring it firmly on the support, shortening the loading time, reducing the negative impact of the support on the active centers, and further improving the polymer morphology, which makes them suitable for use in industrial polymerization temperatures. Herein, we synthesized a α-diimine nickel(II) catalyst bearing four hydroxyl substituents. The hydroxyl substituents enable the catalyst to be immobilized firmly on silica support by covalent linkage in 5–10 min. Compared with the toluene solvent system, the homogeneous catalysts show high activity and thermal stability in hexane solvent at the same conditions. Compared with homogeneous catalysts, heterogeneous catalysis leads to improvements in catalyst lifetime, polymer morphology control, catalytic activity, and the molecular weight of polyethylene (up to 679 kg/mol). The silica-supported catalysts resulted in higher melting temperatures as well as lower branching densities in polyethylenes. Even at 70 °C, the polyethylene prepared by **S-CatA-2** still exhibits dispersed particle morphology, and there is no phenomenon of reactor fouling, which is suitable for industrial polymerization processes.

## 1. Introduction

It is well known that the structure of polyethylene is mainly determined by transition metal catalysts [1]. α-Diimine nickel or palladium complexes have received much attention due to their low oxophilic nature, high tolerance to water and oxygen, and unique “chain-walking” polymerization mechanism for the preparation of branched polyethylene [2,3,4,5]. In this field, researchers have been working on the preparation of highly active and thermally stable homogeneous α-diimine catalysts with well-defined molecular structures and reasonable modifications to play an important role in mechanism research by introducing various substituted groups or modifying the backbone structure of the α-diimine ligand [6,7,8,9,10,11,12,13,14,15,16,17,18,19,20,21,22]. However, homogeneous α-diimine catalysts exhibit shorter lifetimes in the polymerization process and self-decomposition reactions of the M-H active center. Additionally, it is challenging to withdraw the reaction heat generated during polymerization due to the serious reactor fouling caused by a lack of control over the polymer morphology, which limits the application of these catalysts in industry processes. In contrast, the heterogenization of homogeneous α-diimine complexes on supports presents an efficient strategy. It can improve the polymer products morphology, increase the polymer molecular weight, prolong the catalyst lifetime, and meet the requirements of industrial gas-phase or slurry polymerization [23,24,25,26,27,28,29,30,31,32,33].

**Scheme 1:**
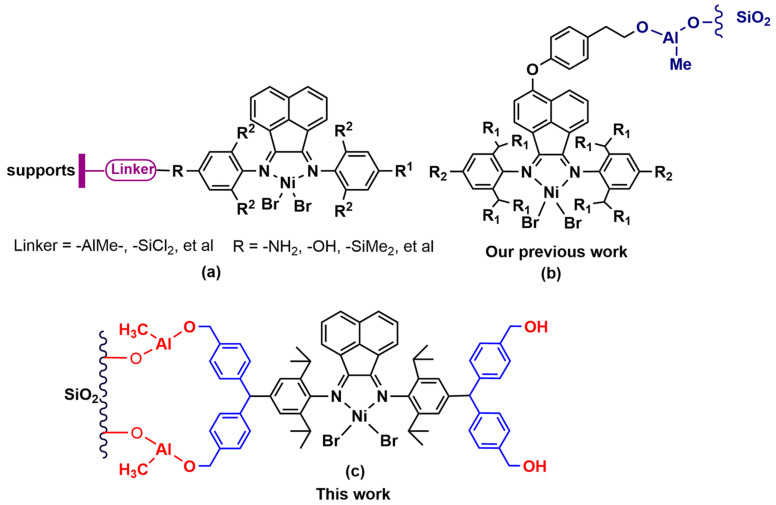
Covalent attachment-supported α-diimine nickel(II) catalysts. (**a**) Other groups’ previous work; (**b**) Our previous work; (**c**) This work.

Covalently attaching the homogeneous catalyst is an efficient way for preventing it from leaching out of the support. The metal complexes can be heterogeneous on support through reacting with a specially designed functional group on the ligand. The interactions of the active group with the support are essential to determine the catalysts’ properties. Brookhart’s team prepared covalently supported α-diimine nickel(II) catalysts (Scheme 1a) by introducing -OH or -NH_2_ functional groups at the *p*-N-aryl position and linking them with TMA-treated SiO_2_, where the supports were covalently bonded to catalysts with specific functional groups to avoid leaching of homogeneous catalysts during polymerization, resulting in polymer exist good morphology [23]. Li et al. modified the allyl-containing α-diimine ligand by introducing reactive Si-Cl end groups, enabling its heterogenization by the reaction of Si-Cl groups with hydroxyl groups on silanol- or ethanolamine-modified Merrifield resins on the silica surface (Scheme 1a) [24]. However, when silica is loaded with Si-Cl-modified ligands, the mixture needs to be refluxed for 48 h, because its catalyst ligands contain fewer Si-Cl end-group reaction sites and less contact with silanols on the silica surface. Chio’s group used SiO_2_ and MgCl_2_ as supports to heterogenize α-diimine nickel(II) catalysts, respectively [25]. The MgCl_2_-supported catalytic system was not as powerful as the SiO_2_-based supported catalytic system, and the resulting polymer morphology was poor; its loading reaction time was up to 15 h. Sun’s group prepared 1-(2,6-dibenzhydryl-4-nitrophenylimino)-2-mesityliminoacenaphthyl nickel bromide catalyst and loaded it on Et_2_AlCl or Et_3_Al_2_Cl_3_ modified silica [26]. The catalyst with the –NO_2_ group reacted with the modified silica for half an hour. The Bernardo-Gusmão’ group prepared a functionalized nickel catalyst, which was attached to mesoporous silica (MCM-41) [27]. But the synthesis reaction of the heterogenized catalyst remained for 2 days. In our previous studies, we developed functional groups on the anthraquinone backbone side of α-diamines as covalently linked α-diimine catalysts reacting with active supports to prepare heterogeneous catalysts (Scheme 1b) [28]. Recently, Chen and coworkers developed a hydrogen-bonding effect and ionic anchoring loading strategy that can significantly improve the thermal tolerance of various olefin polymerization catalysts [29,30,31,32].

For the covalent attachment-supported catalyst, it is necessary to ensure the thermal stability of the catalyst while the catalytic activity is not decreased. The heterogeneous catalysis improves polymer morphology control and avoids reactor fouling. For those reported catalyst structures containing only one or two functional groups, the probability of contact and reaction with the active group on the support is relatively low, and the loading process time is longer. Most of the supported catalysts have lower activity than homogeneous catalysts due to the support’s negative effect. Especially at higher polymerization temperatures, the morphology control of the polymer obtained is poor. Therefore, the important problem is that the active group on the catalyst can quickly react with the support, anchoring it firmly on the support, shortening the loading time, and reducing the negative impact of the support on the active centers.

Herein, we will attempt to design and synthesize a novel α-diimine nickel(II) catalyst that has two benzyl hydroxyl substituents attached to the methylene group of the para-*N*-aryl and isopropyl substituents on the ortho-*N*-aryl (Scheme 1c). In this way, the thermal stability and catalytic performance of the catalyst are improved due to the larger steric hindrance of the ligand. In addition, the four hydroxyl substituents on the ligand can further enhance the reaction probability between the catalyst and the supports by 2–4 times. As a result, the catalyst will be very firmly and quickly attached to the SiO_2_ supports through the covalent linkage strategy, further controlling the morphology of the polymer, avoiding reactor fouling, and shortening the loading process time. The homogeneous catalyst and the two supported catalysts with different nickel loadings were prepared and used in the ethylene polymerization. In the meantime, the influences of reaction conditions and nickel loading of the supported catalyst on the catalytic activity, as well as the branching degree, product melting point, molecular weight, and particle morphology of the polyethylene, were investigated.

## 2. Results and Discussion

### 2.1. Synthesis of α-Diimine Ligand and Complex **CatA**

**Scheme 2:**
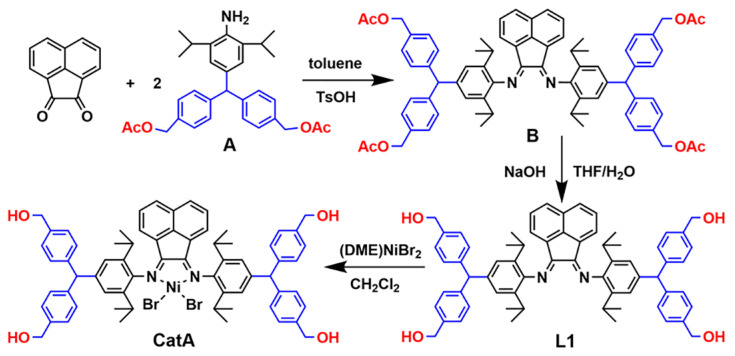
Synthesis process of homogeneous catalyst **CatA**.

It is well known that the classical Brookhart’s catalyst has poor thermal stability. We modified the backbone aniline structure of the ligand to synthesize the α-diimine nickel(II) complex **CatA** (Scheme 2) with isopropyl substituents on the *o*-*N*-aryl and two benzyl hydroxyl substituents attached to the methylene group of the *p-N*-aryl. At first, *N*,*N*-bis[2,6-diisopropylphenyl-4-bis(4-hydroxymethylphenyl)methyl]-acenaphthylene-1,2-diimine ligand **L1** was synthesized. The **CatA** complex was prepared by reacting purified ligand **L1** with (DME)NiBr_2_ in high yield. FT-IR, ^1^H-NMR, and elemental analysis were used to characterize the ligand and the complex.

### 2.2. Ethylene Polymerization with α-Diimine Nickel (II) Complex **CatA**

We used complex **CatA** as the homogeneous catalyst for the ethylene polymerization, and the results are summarized in Table 1. Seeing from Table 1, the polymerization activities of **CatA**/AlEt_2_Cl were higher than 3.00 × 10^6^ gPE/molNi·h at 70 °C under different conditions (Entries 3, 4, 7, 11). More prominently, when the pressure was at 1.0 MPa, the activity of **CatA** was close to 10^7^ gPE/molNi·h at 70 °C. These results thoroughly demonstrate the catalyst’s outstanding temperature stability, which is mainly due to the large steric hindrance of the ligand structure. There are two large benzyl hydroxyl steric hindrance substituents on the p-N-aryl. The cocatalyst anion in the active center ion pair may interact with hydroxyl groups, similarly to our previous catalysts, increasing the distance between the cation species and anion [34,35]. As a result, the insertion of ethylene monomer was easier, and catalytic activity and thermal stability were enhanced. The four hydroxy groups on the ligand are important to improve the catalytic performance.

Generally, homogeneous catalysts exhibit higher activity in toluene solvent and lower activity in n-hexane because of the solubility, so toluene is also used as the polymerization solvent of homogeneous catalysts in most literature. However, we found that the catalyst **CatA** exhibited higher activity in the n-hexane solvent system than the toluene system at the same temperature of 50 °C or 70 °C. At 70 °C, although the activity decreased, it could still reach 6.60 × 10^6^ gPE/molNi·h. We also observed that the polymer obtained in n-hexane showed dispersed powder morphology at 30 °C. On the contrary, the polyethylene obtained in toluene behaved like a rubber-like cluster. This may be due to the four hydroxyl substituents on the complex. The polarity of the complex is relatively large, and the solubility in the non-polar solvent n-hexane is poor; the polymerization process is similar to that of the heterogeneous system. And the poor solubility of the polymer in n-hexane also increases the dispersity of the polymer.

It is also found that under the same conditions, the molecular weight of the polymer obtained in n-hexane (Entries 1 and 3, Table 1) was higher than that obtained in toluene (Entries 5 and 7, Table 1). This is related to the fact that the polarity of n-hexane is smaller than that of toluene. The chain transfer constant of the chain transfer reaction in n-hexane solvent is relatively small, and the probability of chain transfer of the active chain is reduced, which promotes the chain growth reaction. The catalyst **CatA** exhibits high activity and better morphology in n-hexane, and these outstanding advantages enhance the industrial application of this catalyst.

Meanwhile, it can be seen that in the ethylene polymerization, complex **CatA** with AlEt_2_Cl (6.60 × 10^6^ gPE/molNi·h) demonstrates higher activity than when combined with MAO (2.84 × 10^6^ gPE/molNi·h). Therefore, the less expensive AlEt_2_Cl was used as the cocatalyst in further experiments. The molecular weight of the polymer is significantly influenced by polymerization temperature. For example, when the temperature reached 70 °C, the molecular weight of the polymer obtained in n-hexane decreased from 332 kg/mol (30 °C) to 136 kg/mol (Entries 1 and 3, Table 1). The accelerated rate of chain transfer at high temperatures should explain this phenomenon.

**Figure 1:**
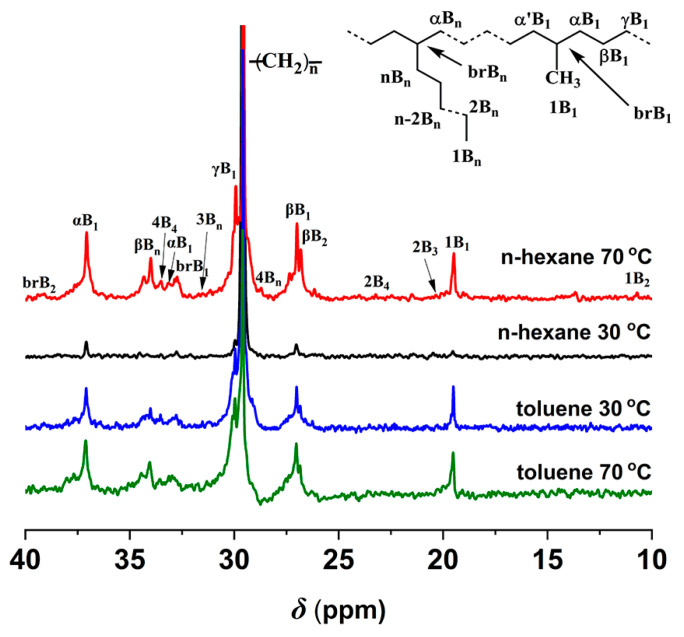
^13^C NMR spectra of polyethylenes prepared by **CatA** (Entries 1, 3, 5, 7 in Table 1).

The polyethylenes were determined by DSC (Figures S5–S7). The melting temperatures (T*_m_*) of the products are shown in Table 1. The melting points of PEs decreased with the temperature raised, regarding the same trends as previously reported α-diimine Ni(II) catalysts. At higher temperatures, the acceleration of chain growth and “chain walking” produced more branches and prevented polymer crystallization [36]. Chain structure plays an essential role in determining the difference in melting point. Further, the chain structures of PEs were characterized by ^13^C NMR spectroscopy (Figure 1), and the results are presented in Table 2.

The branching density was calculated according to the literature [37,38]. The branching density of polyethylene produced by the catalyst **CatA** in n-hexane increased from 35/1000C to 90/1000C when the temperature increased from 30 °C to 70 °C (Entries 1 and 3, Table 2). The resonances of other branch chains increased considerably, with the exception of the methyl chain. Higher polymerization temperatures are more favorable for the occurrence of chain transfer reactions, resulting in more branch chains. With the increase in ethylene pressure, the linear growth reaction of the chain is easier to carry out, which reduced the branching density of the polymer (Entries 3 and 4, Table 2). The solvent has an obvious influence on the branching degree of the resultant polymer, especially at a higher temperature of 70 °C. In the n-hexane solvent system, the branching density of the polymer is 90/1000C (Entry 3, Table 2), which is lower than that of the polymer obtained in the toluene solvent (108/1000C, Entry 7, Table 2). The phenomenon might be explained by the low polarity and low chain transfer constant of n-hexane in comparison to toluene, which reduce the probability of “chain walking”. When the polymerization time was extended from 30 min to 60 min, the yield of polymer was significantly increased at 30 °C or 50 °C, while the yield of polymer increased by only 0.6 g at 70 °C, and the activity was significantly reduced. This indicates that the activity of the homogeneous catalyst declines significantly at high temperatures. Meanwhile, the morphology of the polymer changed from dispersed particles to sticky and clumpy, and the reactor fouling phenomenon became serious with the increase in polymerization temperature. In order to improve these problems, the α-diimine catalyst **CatA** was supported on SiO_2_ through a covalent bond by the reaction of the -OH substituents on the ligand with the activated SiO_2_, to explore the catalytic performance of the heterogeneous catalyst as well as the advantages of the multi-hydroxyl supported sites covalently bonded to the supports strategy with a view to better industrial applicability.

### 2.3. Preparation of Supported α-Diimine Nickel(II) Catalysts (**S-CatA-1, S-CatA-2**)

**Scheme 3:**
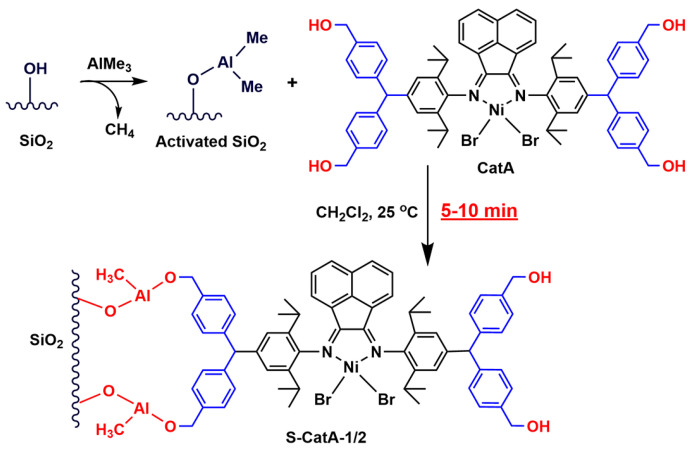
Preparation process of supported catalysts **S-CatA-1/2**.

The silica gel supports were dried for adsorbed moisture at 200 °C, rather than being calcined above 600 °C for several hours as in previous literature, and then treated with trimethylaluminum (TMA). Subsequently, homogeneous α-diimine nickel(II) catalyst **CatA** was reacted with the TMA-modified SiO_2_ supports at room temperature, and the two different nickel content heterogeneous catalysts **S-CatA-1/2** were prepared in dichloromethane (Scheme 3). The homogeneous catalyst solution was added to the suspension of silica gel and stirred for 5 min (**S-CatA-1** and **S-CatA-2** reacted for 10 min, Figure S3). The upper liquid became colorless and transparent quickly after the reaction system stood for one minute, indicating that the upper solvent contained very little homogeneous catalyst. After washing with dichloromethane and drying, the supported catalyst powder with good fluidity and high yield was obtained. The four hydroxyl groups on the catalyst may not all react with the modified support. The structure of the supported catalyst in Scheme 3 only shows one of the possible structures. 

The ICP analysis revealed nickel loading ratios in the supported catalysts. The theoretical nickel loading values of **S-CatA-1/2** on SiO_2_ were 1.00 wt% and 1.50 wt%, respectively, with measured nickel loading values of 0.94 wt% for **S-CatA-1** and 1.41 wt% for **S-CatA-2**. The measured values and the theoretical values are fairly close. This indicates that due to the existence of multiple hydroxyl active groups on the ligand, the homogeneous catalyst **CatA** can efficiently react with the modified support and is very stably supported on the silica gel support. The loading reaction time is greatly reduced from more than an hour in the literature to a few minutes, which is a significant improvement in the loading process of heterogeneous α-diimine catalysts.

**Figure 2:**
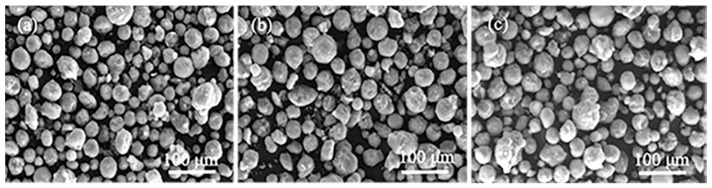
SEM images of SiO_2_ support and **S-CatA-2** heterogeneous catalyst. (**a**) 757#SiO_2_; (**b**) 757#SiO_2_ treated by TMA; (**c**) supported catalyst **S-CatA-2**.

**Figure 3:**
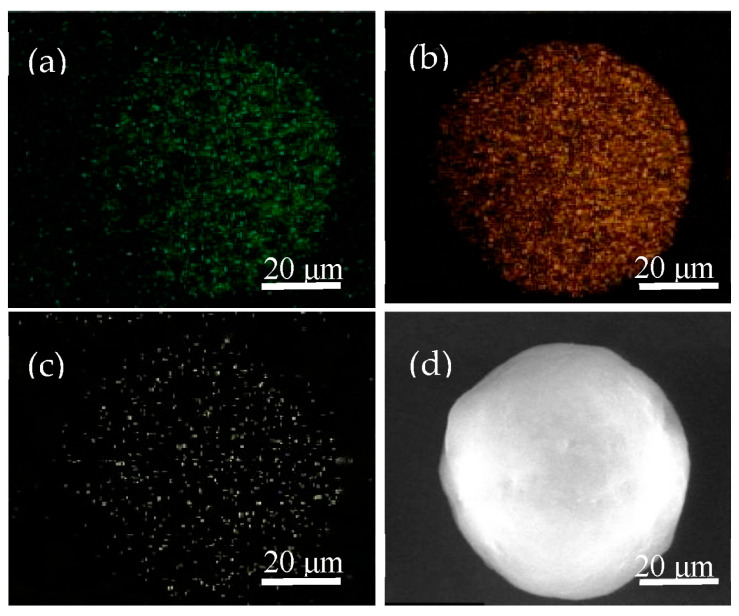
Elemental Al-Si-Ni EDX mapping and SEM images of heterogeneous catalyst **S-CatA-2**. (**a**) distribution of Al element on support; (**b**) distribution of Si element on support; (**c**) distribution of Ni element on support; (**d**) SEM image of **S-CatA-2** catalyst.

**Figure 4:**
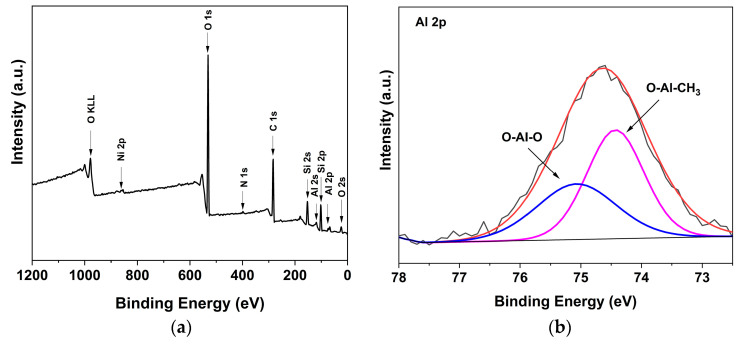
X-ray photoelectron spectroscopy of supported catalyst **S-CatA-2**. (**a**) XPS wide scan spectrum of the **S-CatA-2**; (**b**) Al 2p level spectra of the S-CatA-2.

The SiO_2_ supports and supported catalysts were characterized by SEM. The supported catalyst is similar to the particles of the support and is almost spherical (as shown in Figure 2). Also, we can clearly discover the distribution of elements Al-Si-Ni on the SiO_2_ support surface by EDX mapping of the supported catalyst **S-CatA-2** (Figure 3). Mapping of Al shows a homogeneous distribution of the trimethylaluminum linker used for the attachment of **S-CatA-2**. And mapping of the catalytic metal center was also observed on the surface of silica. We used the supported catalyst **S-CatA-2** to conduct XPS characterization to explore the distinctive connection mode between the supports and the homogeneous catalyst, and the results are shown in Figure 4. The binding energy (BE) peaks of C1s, N1s, Al2p, Ni2p^3^, Si2p, and O1s of the support catalyst appeared at 284, 398, 74, 862, 102, and 531 eV (Figure 4a). Due to the large amount of silicon and oxygen on the SiO_2_ surface, strong absorption signals of Si 2p and O 1s are observed for SiO_2_-supported catalysts. The Al element in the active SiO_2_ supports serves as the “bridge” connecting the supports and the homogeneous catalyst. Figure 4b displays the Al 2p energy level spectrum of the covalently heterogeneous catalyst. The absorption of Al 2p occurs at about 75.03 eV and 74.45 eV, which have been attributed to O-Al-O and O-Al-CH_3_, respectively [39]. The analysis mentioned above illustrates that complex **CatA** has been successfully attached to SiO_2_ supports through the covalent bond.

### 2.4. Ethylene Polymerization with Supported Catalysts (**S-CatA-1**, **S-CatA-2**)

The results of ethylene slurry polymerizations using supported catalysts **S-CatA-1** and **S-CatA-2** with AlEt_2_Cl in n-hexane are summarized in Table 3. Compared with the catalytic activity of homogeneous catalysts, when the polymerization temperature is 70 °C, the catalytic activity of lower loading supported catalyst **S-CatA-1** is comparable to that of homogeneous catalysts. However, the activity of the higher loading-supported catalyst **S-CatA-2** was 4.07 × 10^6^ gPE/molNi·h (Entry 7, Table 3), while the activity of the catalyst **CatA** was 3.90 × 10^6^ gPE/molNi·h (Entry 10, Table 1). The activity of the supported catalyst **S-CatA-2** did not decrease; it was even slightly higher than that of the homogeneous catalyst under the same conditions. The two supported catalysts, **S-CatA-1** and **S-CatA-2,** with different nickel loadings show good activity and thermal stability. This may be due to the fact that the hydroxyl group on the homogeneous catalyst **CatA** for reaction with the supports is far from the active center, thereby reducing the negative effect of the support on the active centers. The high activity and stable catalytic active centers are formed through the covalent bonding of its hydroxyl group to the support.

**Figure 5:**
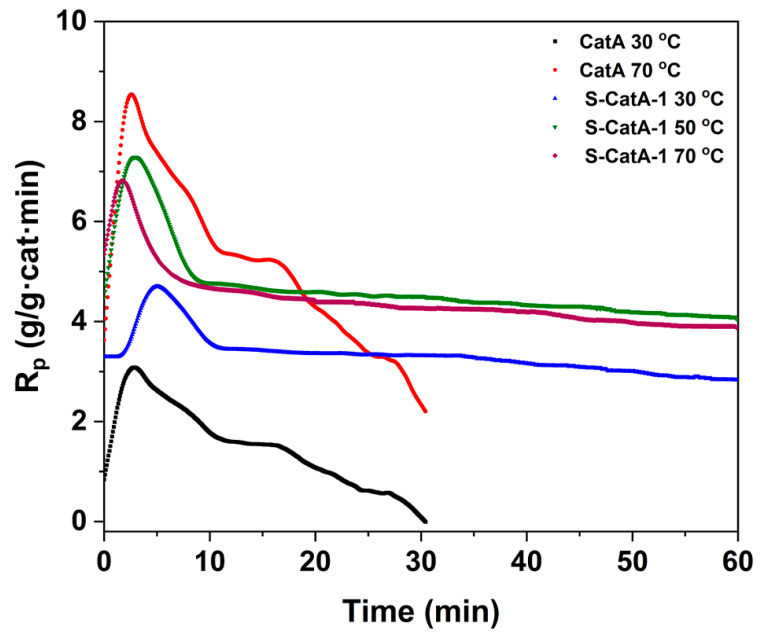
The kinetic curves of ethylene polymerization.

It was obvious from the kinetic plots of polymerization (Figure 5) that the catalytic reaction of homogeneous catalyst **CatA** exhibits a high initial rate followed by a rapid decay, a typical decay type. After the initial high polymerization rate, the system-supported catalyst **S-CatA-1** maintains a stable polymerization rate until the reaction is completed within an hour. We believed that the connecting silica gel to the homogeneous catalyst would not only inhibit the insertion of ethylene due to the steric hindrance of the Ni complex, but also delay the catalyst’s deactivation, extending its lifetime. This entirely demonstrates that the catalytic activity of our supported catalyst is very stable and suitable for industrial polymerization processes.

**Figure 6:**
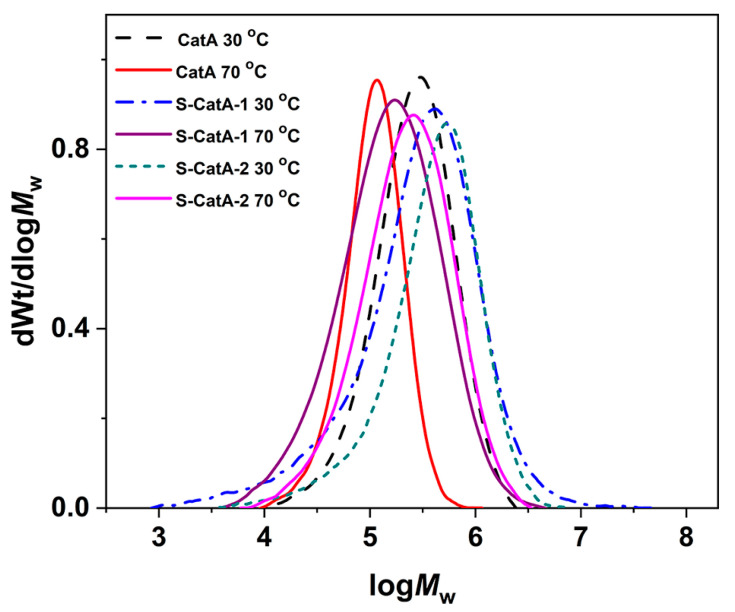
GPC curves of resultant polyethylenes (Entries 8, 10 in Table 1, Entries 1, 3, 5, and 7 in Table 3).

In comparison with the homogeneous catalyst, notable higher molecular weight polymers were produced by the supported catalysts (Figure 6). For instance, at 30 °C, the molecular weights of resultant PEs prepared from the two supported catalysts **S-CatA-1**/**2** were 503 kg/mol and 679 kg/mol, respectively (Entries 1 and 5, Table 3), which were higher than those obtained from the homogeneous catalyst **CatA** (359 kg/mol, Entry 8, Table 1) under the same conditions (Figure S10). The higher molecular weight can be explained by reduced chain termination and/or higher chain propagation. The steric hindrance of the support effectively inhibits the chain transfer rates and promotes the chain propagation reaction as well. With the increase in ethylene pressure from 0.5 MPa to 1.0 MPa, the molecular weight of the polyethylene increased in the two catalytic systems.

**Figure 7:**
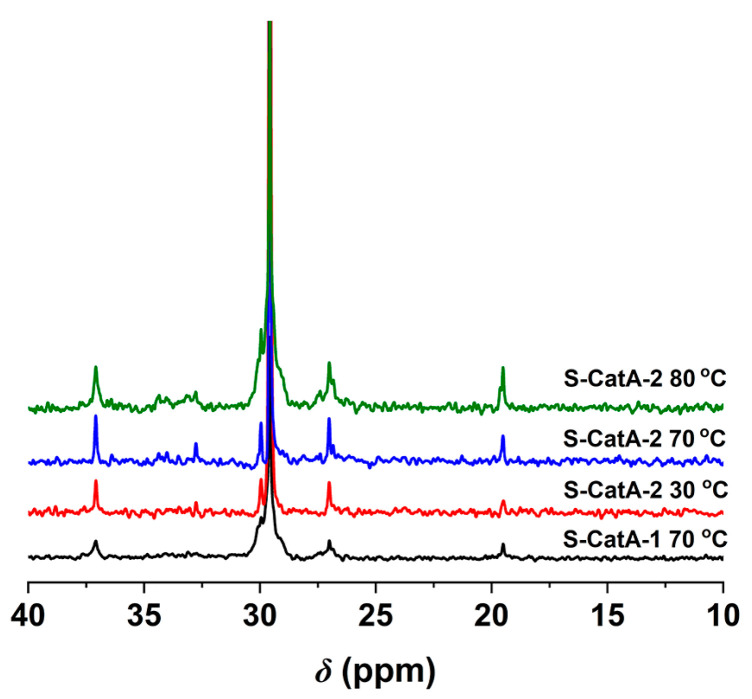
^13^C NMR spectra of polyethylenes (Entries 3, 5, 7, and 8 in Table 3).

Whereas Figure 7 and Table 4 indicate that the degree of branching in polyethylene obtained with the **S-CatA-1/2** catalyst was significantly reduced. At 70 °C, the polyethylene obtained from **S-CatA-2** possessed 52/1000C branches, involving methyl (61.1%), ethyl (10.7%), propyl (4.2%), butyl (5.8%), pentyl (4.5%), and LCB (13.7%). The melting points of polyethylene obtained from **S-Cat-1/2** are higher than 120 °C (Figures S8 and S9). This could be explained by the fact that the rate of β-H elimination reactions and “chain walking” during polymerization is hindered by the spatial positional resistance of the support. The degree of branching of polyethylene prepared from **S-CatA-2** catalyst increased from 34/1000C to 67/1000C as the temperature increased from 30 °C to 80 °C, accompanied by a percentage reduction of methyl branched chains and an increase of long branched chains. This is consistent with the results of reported catalytic systems [15,34,35].

We further investigated the effect of nickel loading on catalytic activity and the resulting polymer. Firstly, comparing the catalytic performance of two catalysts, **S-CatA-1** and **S-CatA-2,** with different nickel loadings, the catalytic activity of **S-CatA-2** is higher than that of **S-CatA-1** under the same polymerization conditions (Table 3). The catalytic activities of **S-CatA-1** and **S-CatA-2** were 4.02 × 10^6^ gPE/molNi·h and 6.92 × 10^6^ gPE/molNi·h at 50 °C, respectively (Entries 2 and 6, Table 3) (Figure S11). In the catalytic system **S-CatA-2** with a higher nickel loading rate, more nickel metal centers will be converted into cationic active centers during the polymerization process, thus more active centers will be exposed, which increases the probability of collision and reaction between ethylene monomers and active centers, and more polymers will be obtained.

The nickel loading of the catalyst also significantly affects the chain structure and molecular weight of the polymer. Table 3 and Table 4 show that supported catalyst **S-CatA-2** produced polyethylenes with higher molecular weights as well as lower branching densities than catalyst **S-CatA-1**. For instance, at 70 °C, the polyethylene obtained from **S-CatA-2** exhibited a 52/1000C branching density and a molecular weight of 244 kg/mol, while the branching density of the polyethylene obtained from **S-CatA-1** was 63/1000C, and the molecular weight was 190 kg/mol (Figure S12). We know that after the nickel loading increased, the space of the nickel center on the supports is relatively crowded, and the “chain walking” and chain transfer reactions on the active centers are more easily restricted, resulting in a decrease in branching density and an increase in molecular weight.

### 2.5. Morphology of Polymers Obtained over Homogeneous and Heterogeneous Catalytic Systems

**Figure 8:**
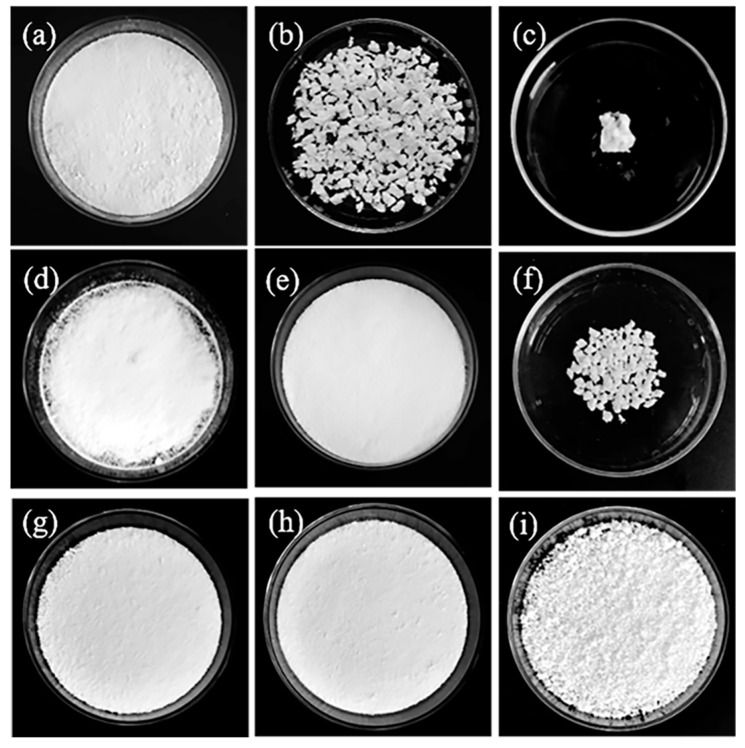
Photographs of resultant polyethylenes (**a**), **CatA** 30 °C in n-hexane; (**b**), **CatA** 50 °C in n-hexane; (**c**), **CatA** 30 °C in toluene; (**d**), **S-CatA-1** 30 °C; (**e**), **S-CatA-1** 50 °C; (**f**), **S-CatA-1** 70 °C; (**g**), **S-CatA-2** 30 °C; (**h**), **S-CatA-2** 50 °C; (**i**), **S-CatA-2** 70 °C.

**Figure 9:**
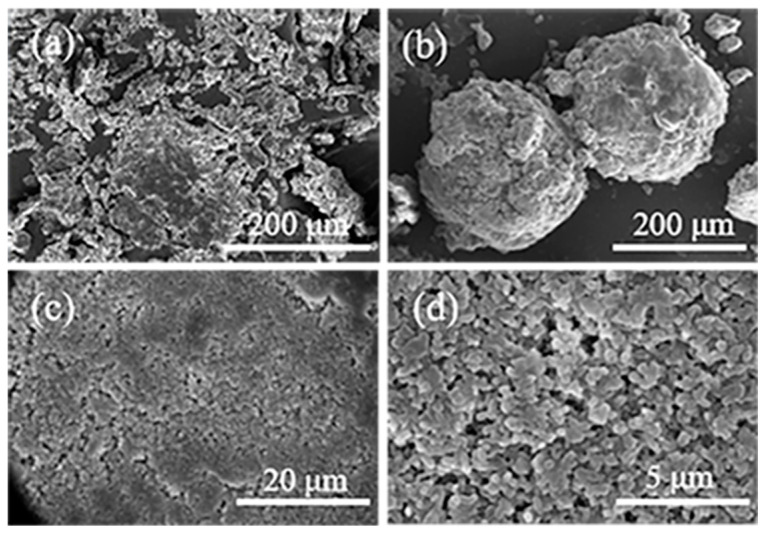
SEM images of resultant polyethylenes (**a**) **CatA** 30 °C; (**b**–**d**) **S-CatA-2** 50 °C.

In addition to the catalytic performance parameters mentioned above (activity, stability, and polymer microstructure), the morphology of polymers is equally important in evaluating catalyst performance and whether it is suitable for industrial polymerization processes. We compared the appearance morphology of the polymers obtained with different catalysts, as shown in Figure 8. The polymer obtained with homogeneous catalysts in n-hexane at 30 °C is dispersed in powder form (Figure 8a). As the polymerization temperature increased, the dispersed morphology of the polymer became worse and stuck together due to the increase in the branching degree of the polymer (Figure 8b,c). On the contrary, at 30 °C and 50 °C, the polymers obtained from supported catalysts **S-CatA-1** and **S-CatA-2** show the free-flowing morphology (Figure 8d,e,g,h), which is obviously different from the morphology of polyethylene prepared by homogeneous catalysts. The polyethylene prepared by **S-CatA-2** still exhibits dispersed particle morphology at 70 °C, and reactor fouling caused by the leaching of catalyst **CatA** from the silica supports does not occur. Although the product obtained from the homogeneous catalyst shows good dispersion at 30 °C, the SEM image of the polyethylene shows that it has a powdery microstructure with no particle morphology (Figure 9a). In contrast, the polymer obtained by a silica-supported catalyst clearly replicates the morphology of the support, showing a spherical particle shape (Figure 9b). Magnification-based observations of the accumulation of roughly spherical polymer sub-particles are presented (Figure 9d).

The bulk densities of polyethylene products prepared with supported catalysts **S-CatA-1**/**2** at 30 °C and 50 °C were measured. At 30 °C and 50 °C, the bulk densities of polyethylenes obtained from catalyst **S-CatA-1** were 0.32 and 0.24 g/cm^3^, respectively. The bulk densities of polyethylene products prepared by catalyst **S-CatA-2** were 0.28 and 0.23 g/cm^3^. This further demonstrates that the supported catalyst can prepare polyethylene products with excellent morphology and good flowability, providing the possibility for gas-phase or slurry industrial polymerization and the advantage of the multi-hydroxy substituent covalent connection strategy.

## 3. Materials and Methods

### 3.1. General Methods and Materials

The reactions of the air- and/or moisture-sensitive materials were performed in a dry, purified Ar atmosphere using the standard Schlenk technique. The required solvents were dried through activated-4 Å molecular sieves, and then refluxed with CaH_2_ or Na in distillation in an Ar atmosphere. Methylaluminoxane (MAO, 1.5 mol/L in toluene), diethylaluminum chloride (AlEt_2_Cl, 1.0 M in n-hexane) and trimethylaluminum (1.0 M in n-hexane) were purchased from Yanfeng Technology Co., Ltd. (Shenyang, China). The 2,6-diisopropyl-4-bis [4-(methyl acetate)phenyl] methylaniline was purchased from Zhengzhou Te Li Kai Chemical Technology Co., Ltd. (Zhengzhou, China). The 757# SiO_2_ was supplied by Daqing Chemical Research Center (Daqing, China) and calcined at 200 °C in a muffle furnace before use. The surface area of 757# SiO_2_ is 262 m^2^/g, the pore volume is 1.4 cm^3^/g, and the pore diameter is 17.9 nm. The purity of 99.9% ethylene was purchased from Sematic Specialty Gases Co., Ltd. (Tianjin, China). Other chemicals and reagents were used as received.

### 3.2. Characterizations

FT-IR spectra of the ligand and corresponding nickel complex were obtained by pressing KBr pellets using a Thermo Nicolet 6700 spectrometer (Thermo Fisher Scientific, Waltham, MA, USA). The Flash EA 1112 microanalyzer (Thermo Fisher Scientific, Waltham, MA, USA) was used for the elemental analysis. ^1^H NMR spectra of intermediate compounds and ligands were documented at ambient temperature in CDCl_3_ using TMS as an internal standard on a Bruker DMX 400 MHz instrument (Bruker Co., New Castle, DE, USA); δ values were given in ppm and J values in Hz. Through the use of an inductively coupled plasma (ICP) atomic emission spectrometer (ICP-715ES) (Thermo Jarrell Ash Co., Waltham, MA, USA), the nickel loading of supported catalysts was determined. On a Thermo Scientific ESCALAB Xi+, X-ray photoelectron spectroscopy (XPS) (Thermo Fisher Scientific, Waltham, MA, USA) of supported catalysts was performed. Scanning electron microscopy (SEM) was performed on a Novanos SEM 450 (FEI Co., Hillsboro, OR, USA) to analyze the morphology of the supported catalysts and polyethylene. The distribution of Si, Al, and Ni elements on the supported catalyst was characterized using EDX. The branched chain structure of polyethylene was tested on a Bruker DMX 400 MHz instrument (Bruker Co., New Castle, DE, USA) at 120 °C in deuterated 1,2-dichlorobenzene, with TMS used as an internal standard. The δ values were given in ppm. The molecular weight and molecular weight distribution of products were determined using PL-GPC-220 (Agilent Technologies, Inc., Santa Clara, CA, USA) with 1,2,4-trichlorobenzene as solvent at 150 °C. Differential scanning calorimetry (DSC) measurements were performed on a TA Instruments DSC Q20 (PerkinElmer, Inc., Waltham, MA, USA). The melting temperature (*T*_m_) values were obtained in the second heating run at 10 °C/min under an N_2_ atmosphere. The degree of crystallinity (*χ*_c_, %) was calculated from the heat of fusion (Δ*H*_f_/Δ*H*_f_^°^) × 100%, where Δ*H*_f_^o^ is the heat of fusion of folded-chain polyethylene (289.0 J/g) [40]. The bulk density of polyethylene was determined with the standard method ASTM D1895-69 [41]. The polymerization activity equation is: A_1_ = m/(n × t), A_2_ = m/(m_c_ × t). Where m is the mass of the polymerization product, g; m_c_ is the mass of the support catalyst in the polymerization reaction, g; n is the mole amount of the catalyst in the polymerization experiment, mol; and t is the polymerization reaction time, h.

### 3.3. Preparation of α-Diimine Nickel (II) Complex

Synthesis of *N*,*N*-bis[2,6-diisopropylphenyl-4-bis(4-hydroxymethylphenyl)methyl]-acenaphthylene-1,2-diimine ligand **L1**.

A mixture of acenaphthenequinone (1 g, 5.49 mmol), 2,6-diisopropyl-4-bis[4-(methylacetate)phenyl]methylaniline compound A (6.69 g, 13.72 mmol), and an amount of *p*-toluene-sulfonic acid in toluene (150 mL) was refluxed at 180 °C for 10 h. After the reaction, the product was concentrated by cooling, extracted with CH_2_Cl_2_, dried with anhydrous Na_2_SO_4_ and purified by column chromatography to give *N*,*N*-bis[2,6-diisopropylphenyl-4-bis(4-benzylacetate)methyl]-acenaphthylene-1,2-diimine compound B. ^1^H-NMR (400 MHz, CDCl_3_, δ, ppm): δ 7.91 (d, *J* = 8.4 Hz, 2 H), δ 7.38 (d, *J* = 7.6 Hz, 2 H), δ 7.34 (d, *J* = 8.0 Hz, 8 H), δ 7.22 (d, *J* = 8.0 Hz, 8 H), δ 7.00 (s, 4 H), δ 6.65 (d, *J* = 7.2 Hz, 2 H), δ 5.62 (s, 2 H), δ 5.13 (s, 8 H), δ 3.02–2.92 (m, 4 H), δ 2.12 (s, 12 H), δ 1.13 (d, *J* = 6.8 Hz, 12 H), δ 0.87 (d, *J* = 6.8 Hz, 12 H). MS (MALDI-TOF): *m*/*z* 953 (M + H).

The dried 250 mL aubergine flask was replaced with argon three times. Under an argon atmosphere, 20 mL of tetrahydrofuran solution of compound B (0.5 g, 0.45 mmol) and 20 mL of distilled water solution of NaOH (0.3 g, 7.55 mmol) were sequentially added, stirred, and reacted at 120 °C for 3 h. The product was concentrated by cooling, extracted with ethyl acetate, and then dried over anhydrous Na_2_SO_4_, and the resulting crude product was separated by column chromatography to obtain pure *N*,*N*-bis[2,6-diisopropylphenyl-4-bis(4-hydroxymethylphenyl)methyl]-acenaphthylene-1,2-diimine **L1**. FT-IR (ν, cm^−1^): 3260, 3051, 3014, 2962, 2919, 2868, 2714, 2588, 1910, 1794, 1659 (C = N), 1598 (C = N), 1511, 1451, 1328, 1269, 1212, 1174, 1101, 1018, 935, 869, 828, 792, 696, 617, 578. Elemental analysis C_66_H_68_O_4_N_2_ (953.26) (%): Theoretical values: C, 83.16; H, 7.19 N, 2.94. Measured values: C, 83.08; H, 7.28; N, 2.89. ^1^H-NMR (400 MHz, CDCl_3_, δ, ppm): δ 7.92 (d, *J* = 8.0 Hz, 2 H), δ 7.37 (d, *J* = 8.0 Hz, 8 H), δ 7.24 (d, *J* = 8.0 Hz, 8 H), δ 7.02 (s, 4 H), δ 6.67 (d, *J* = 7.2 Hz, 2 H), δ 5.65 (s, 2 H), δ 4.75 (s, 8 H), δ 3.04–2.94 (m, 4 H), δ 1.14 (d, *J* = 6.8 Hz, 12 H), δ 0.88 (d, *J* = 6.8 Hz, 12 H) (Figure S1). MS (MALDI-TOF): *m*/*z* 953 (M + H) (Figure S2).

Synthesis of N, N-bis[2,6-diisopropylphenyl-4-bis(4-hydroxymethylphenyl)methyl]-acenaphthylene-1,2-diimine nickel dibromide (**CatA**).

*N*,*N*-bis[2,6-diisopropylphenyl-4-bis(4-hydroxymethylphenyl)methyl]-acenaphthylene-1,2-diimine ligand L1 (1.1 mmol) and (DME)NiBr_2_ (1 mmol) were dissolved in 40 mL of dichloromethane in a three-necked flask filled with argon gas. Then the reaction mixture was stirred at 40 °C and refluxed for 48 h. The solvent was pulled out, then the product was washed with n-hexane for 3 times before being vacuum dried to obtain the orange-brown powder **CatA** in an 86% yield. FT-IR (ν, cm^−1^): 3415, 3053, 3014, 2958, 2922, 2864, 1799, 1647 (C = N), 1587 (C = N), 1508, 1456, 1421, 1377, 1292, 1261, 1201, 1097, 1016, 952, 821, 779, 684, 615, 532. Elemental analysis: C_66_H_68_Br_2_N_2_NiO_4_ (1171.78) (%): Theoretical values: C, 67.65; H, 5.85; N, 2.39. Measured values: C, 67.72; H, 5.79; N, 2.33.

Due to the fact that the complex nickel element causes paramagnetic problems in NMR tests, the complex was characterized by elemental analysis and FT-IR tests. Seeing from the IR spectra of the ligand **L1** and **CatA**, it can be found that the C = N bond absorption peak of the ligand **L1** is attributed at 1659 cm^−1^ and 1598 cm^−1^, while the C = N bond absorption peak of the complex **CatA** is attributed at 1647 cm^−1^ and 1587 cm^−1^ (Figure S4). Compared with the ligand, the C = N bond absorption peaks of the complex are obviously blue-shifted, so it can be considered that the metal center Ni is coordinated with the ligand, and the results of the elemental analysis tests show that the target product **CatA** was prepared.

### 3.4. Synthesis of Supported Catalysts **S-CatA-1** and **S-CatA-2**

The silica gel (1 g, 757#, calcined at 200 °C) and 40 mL of n-hexane and trimethylaluminum (1 M in n-hexane, 3 mL) were sequentially added into a three-necked flask with mechanical stirring for 15 min at 0 °C, and 5 h at 45 °C. The excess solution was taken out after the reaction. The activated SiO_2_ was washed several times with n-hexane before even being filtered and vacuum-dried at 20 °C for 10 h.

At room temperature, CH_2_Cl_2_ solution of α-diimine nickel(II) **CatA** catalyst was added to a certain amount of activated SiO_2_ supports in CH_2_Cl_2_ solution under mechanical stirring (stirring speed of 100 r/min) for 5–10 min. The reaction system stood for one minute until the upper layer of the solution became transparent. Then, the excess solution was removed, and the remaining part was washed three times with CH_2_Cl_2_ and dried under vacuum to the obtain red-brown supported catalyst powders **S-CatA-1** (94% yield, theoretical nickel loading 1.00%, measured nickel loading 0.94%) and **S-CatA-2** (93% yield, theoretical nickel loading 1.50%, measured nickel loading 1.41%) with good flowability. The silica supports and supported catalysts were characterized by SEM. Taking the supported catalyst **S-CatA-2** as an example, it was characterized by X-ray photoelectron spectroscopy.

### 3.5. Ethylene Polymerization

In a 100-mL stainless steel vessel with a temperature-pressure control system and a magnetic stirrer, polymerizations were carried out. The reactor was dried for two hours in a vacuum at 100 °C, cooled to room temperature in an Ar environment, and then purged three times with dry Ar and once with ethylene. Following the sequential injection of the solvent, cocatalyst solution, and a specific volume of catalyst solution, the polymerization reaction was sustained at the accurate temperature and ethylene pressure for 30 or 60 min. When cooling the reaction temperature to 25 °C, the mixture was poured into the 10 vol% HCl/C_2_H_5_OH solution. The products were alternately washed in C_2_H_5_OH and water and dried in a vacuum oven at 60 °C for 8 h.

## 4. Conclusions

In summary, the novel α-diimine nickel(II) catalyst with isopropyl substituents on the *o*-*N*-aryl group and two benzyl hydroxyl substituents attached to the methylene group of the *p*-*N*-aryl was synthesized and used in ethylene polymerization with n-hexane or toluene as solvent. The homogeneous catalyst **CatA** exhibited higher activity, a higher molecular weight of the polymer, a lower branching degree, and a better morphology of the polymer in n-hexane than in toluene solvent. With the increase in temperature from 30 °C to 70 °C in n-hexane, the branching degree of the resultant polyethylene increased from 35/1000C to 90/1000C. 

In the presence of four hydroxyl substituents, the homogeneous catalyst can be very firmly loaded onto the silica supports by covalent linkage in 5–10 min. It is understandable from the polymerization kinetic curve that the catalyst’s lifetime is prolonged and that the particle morphology is generally superior to that of the homogeneous system. The activities of the silica-supported catalyst **S-CatA-2** and the molecular weights of the polymers were higher than those of a homogeneous catalyst at the same conditions. The catalytic activity of **S-CatA-2** reached 4.51 × 10^6^ g/mol Ni·h at 70 °C and 1 MPa ethylene pressure. The increase in nickel loading from 0.94 to 1.41 wt% results in an increase in productivity on a kg PE/g supported catalyst basis. The melting points, molecular weights, branching degrees, and bulk densities of polyethylene can be varied by the nickel loading of the supported catalyst and polymerization conditions. The advantages, such as shorter loading times, steady-state kinetic profiles, high activity and thermal stability, adjustability of polymer properties, and good morphology of polymers, make our supported catalyst more suitable for gas-phase and slurry industrial polymerization processes. The exploration of further improving the activity of the supported catalyst at higher temperatures by increasing the steric hindrance of the ligand is in progress.

## Figures and Tables

**Table 1:** The results of ethylene polymerization with the homogeneous catalyst **CatA** ^a^.

Entry	*T* °C	*P* MPa	*t* min	Yield g	Act. ^c^	*T*_m_ ^d^ °C	*χ*_c_ ^e^ (%)	*M*_w_ ^e^ kg/mol	PDI ^e^
1	30	0.5	30	0.89	1.77	120	16	332	2.9
2	50	0.5	30	3.84	7.68	107	6	n.d.	n.d.
3	70	0.5	30	3.30	6.60	94	2	136	2.8
4	70	1.0	30	4.73	9.46	110	5	439	2.3
5 ^b^	30	0.5	30	1.02	2.04	119	38	141	2.3
6 ^b^	50	0.5	30	2.73	5.46	105	5	n.d.	n.d.
7 ^b^	70	0.5	30	1.98	3.96	74	1	114	2.4
8	30	0.5	60	2.60	2.60	118	28	359	2.4
9	50	0.5	60	6.51	6.51	n.d.	n.d.	n.d.	n.d.
10	70	0.5	60	3.90	3.90	95	2	152	2.1
11 ^f^	70	0.5	30	1.42	2.84	n.d.	n.d.	n.d.	n.d.

^a^ Condition: n(Ni) = 1 μmol, n(Al)/n(Ni) = 1000, V(n-hexane) = 50 mL. ^b^ V(toluene) = 50 mL. ^c^ Activity = 10^6^ gPE/molNi·h. ^d^ Determined by DSC. ^e^ Determined by GPC. n.d. = not determined. ^f^ cocatalyst: MAO.

**Table 2:** Distribution of polyethylene branches obtained with **CatA** ^a^.

Entry	*T* °C	*P* MPa	Branches (1000 C)	Percentage over Total Branching (%)					
				Methyl	Ethyl	Propyl	Butyl	Amyl	LCB ^b^
1	30	0.5	35	80.8	9.1	0.9	0	0	9.2
3	70	0.5	90	63.5	11.3	9.0	0	0.8	15.4
4	70	1.0	82	70.9	9.8	1.0	4.7	0.6	13.0
5 ^c^	30	0.5	41	83.4	4.9	1.7	2.2	0.7	7.1
7 ^c^	70	0.5	108	78.9	4.6	1.5	3.0	1.5	10.5

^a^ Measured by ^13^C NMR spectroscopy; ^b^ LCB: long chain branching (≥C6); ^c^ toluene solvent.

**Table 3:** Results of ethylene polymerization catalyzed by **S-CatA-1/2** catalysts ^a^.

Entry	Catalyst	Ni ^b^ wt%	*T* °C	Act. ^c^	Act. ^d^	*T*_m_ ^e^ °C	*χ*_c_ ^e^ (%)	*M*_w_ ^f^ kg/mol	PDI ^f^
1	S-CatA-1	0.94	30	2.12	353	130	39	503	4.6
2	S-CatA-1	0.94	50	4.02	641	125	23	n.d.	n.d.
3	S-CatA-1	0.94	70	3.86	615	120	13	190	3.0
4 ^g^	S-CatA-1	0.94	70	4.28	681	125	19	205	4.3
5	S-CatA-2	1.41	30	3.72	892	131	41	679	3.3
6	S-CatA-2	1.41	50	6.92	1656	128	24	n.d.	n.d.
7	S-CatA-2	1.41	70	4.07	982	121	14	244	2.7
8	S-CatA-2	1.41	80	1.38	265	120	9	n.d.	n.d.
9 ^g^	S-CatA-2	1.41	70	4.51	1079	123	16	449	4.6

^a^ Condition: n(Ni) = 1 μmol, n(Al)/n(Ni) = 1000, V(n-hexane) = 50 mL, t = 60 min. ^b^ The nickel loading is determined by ICP. ^c^ Activity, 10^6^ gPE/molNi·h. ^d^ Activity, gPE/gcat·h. ^e^ Determined by DSC. ^f^ Determined by GPC. ^g^ P_Et_ = 1.0 MPa. n.d. = not determined.

**Table 4:** Distribution of polyethylene branches obtained with **S-CatA-1/2** ^a^.

Entry	Catalyst	*T* °C	*P* MPa	Branches (1000C)	Percentage over Total Branching (%)					
					Methyl	Ethyl	Propyl	Butyl	Amyl	LCB ^b^
3	**S-CatA-1**	70	0.5	63	68.6	9.2	6.2	1.1	1.3	13.6
4	**S-CatA-1**	70	1.0	55	69.0	8.1	2.4	3.2	1.4	15.9
5	**S-CatA-2**	30	0.5	34	68.9	13.8	3.1	2.0	2.3	9.9
7	**S-CatA-2**	70	0.5	52	61.1	10.7	4.2	5.8	4.5	13.7
8	**S-CatA-2**	80	0.5	67	57.8	11.4	3.7	3.8	3.1	20.2

^a^ Measured by ^13^C NMR spectroscopy; ^b^ LCB: long chain branching (≥C6).

## Data Availability

The authors declare data availability.

## Supplementary Materials

The supporting information can be downloaded at: https://www.mdpi.com/article/10.3390/ijms241713645/s1.
