# Supplementary material for: Synthesis of α-Diimine Complex Enabling Rapidly Covalent Attachment to Silica Supports and Application of Homo-/Heterogeneous Catalysts in Ethylene Polymerization
Source: Int J Mol Sci. 2023 Sep 4;24(17):13645. doi: 10.3390/ijms241713645 (PMC10487567; doi:10.3390/ijms241713645)
Supplement: Supplementary file 1 [file ijms-24-13645-s001.zip › ijms-2558098-supplementary.pdf]

Supplementary Information

Synthesis of α-Diimine Complex Enabling Rapidly Covalent Attachment to Silica Supports and Application of Homo-/Heterogeneous Catalysts in Ethylene Polymerization

Xiaobei Zhao [1], Yanhui Hou [2,*], Linlin Ye [1], Kening Zong [1], Qingming An [2], Binyuan Liu [1] and Min Yang [1,*]

1 Hebei Key Laboratory of Functional Polymers, Institute of Polymer Science and Engineering, Hebei University of Technology, Tianjin 300130, China
2 State Key Laboratory of Separation Membranes and Membrane Processes, School of Material Science and Engineering, Tiangong University, Tianjin 300160, China
* Correspondence: houyh1977@163.com (Y.H.); polyym@163.com (M.Y.)

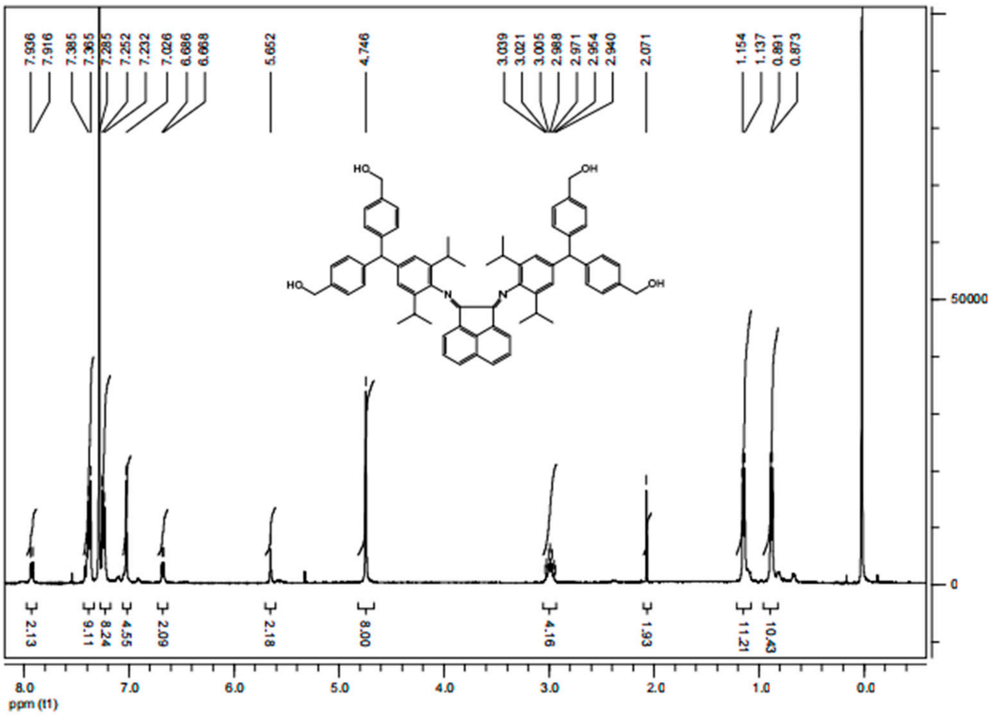

Figure S1 The ^{1}H NMR spectrum of **L1**

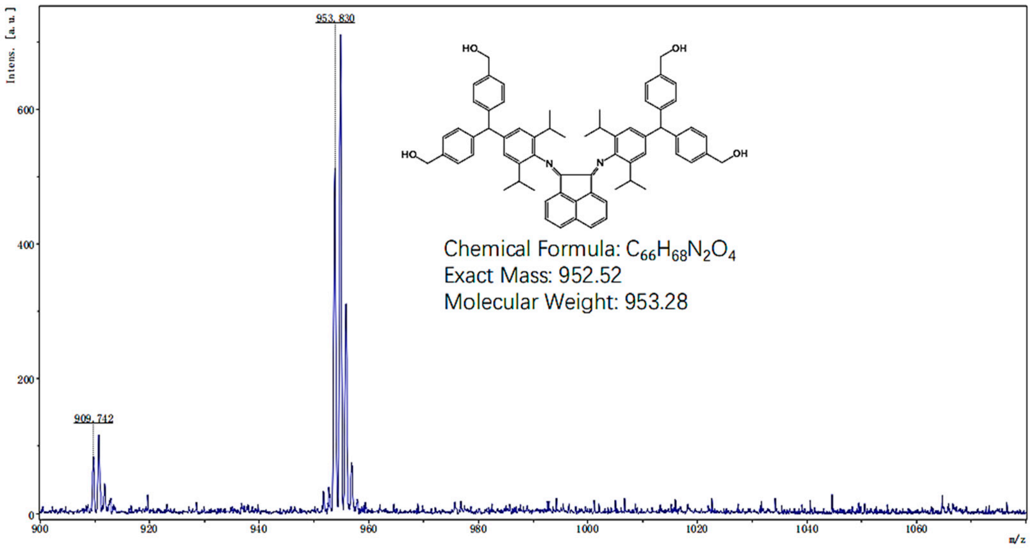

Figure S2 The MS(ESI) spectrum of **L1**

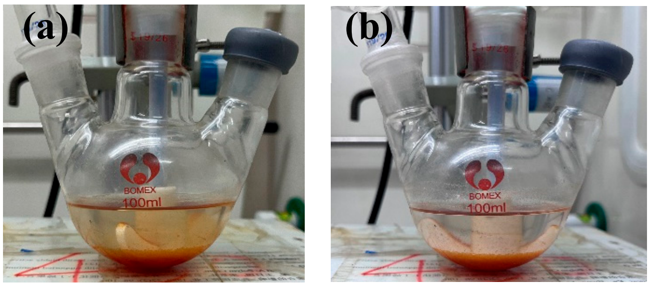

Figure S3 Preparation process of supported catalyst **S-CatA-1**

(a) Reaction of 1 minute; (b) Reaction of 5 minutes

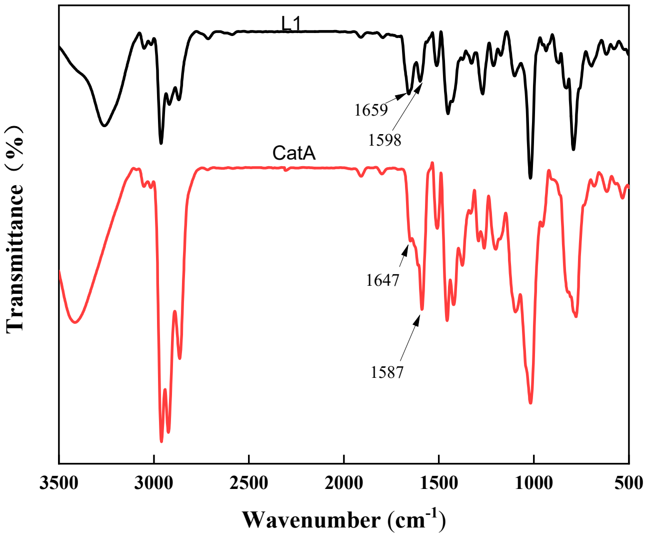

FigureS4 FT-IR spectra of **L1** and corresponding complex **CatA**

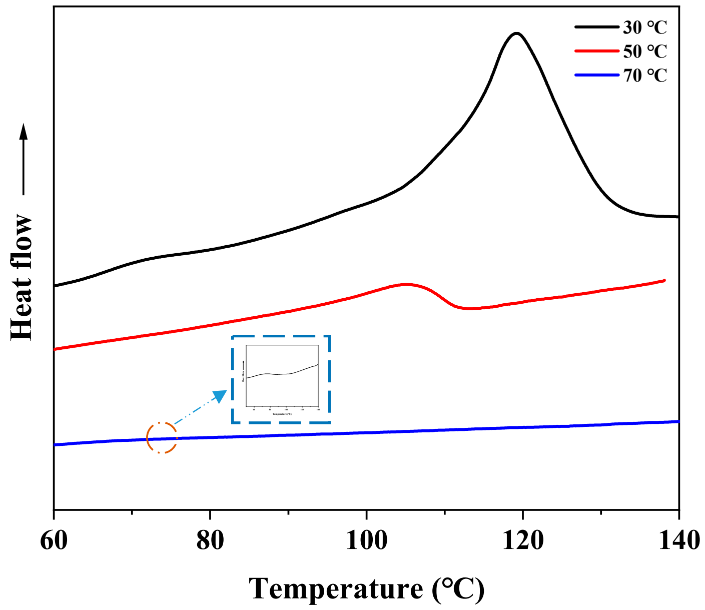

FigureS5 DSC curves of polyethylenes prepared by **CatA** in toluene solvent (Entries 5, 6, 7 in Table 1)

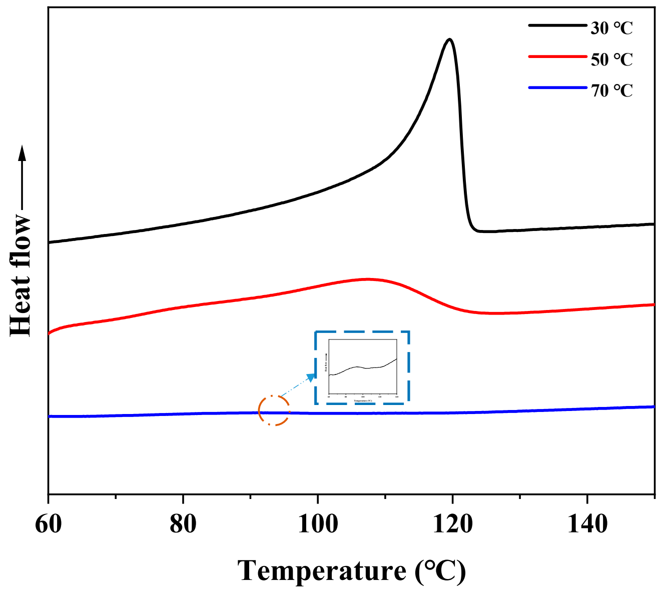

FigureS6 DSC curves of polyethylenes prepared by **CatA** in n-hexane solvent (Entries 1, 2, 3 in Table 1)

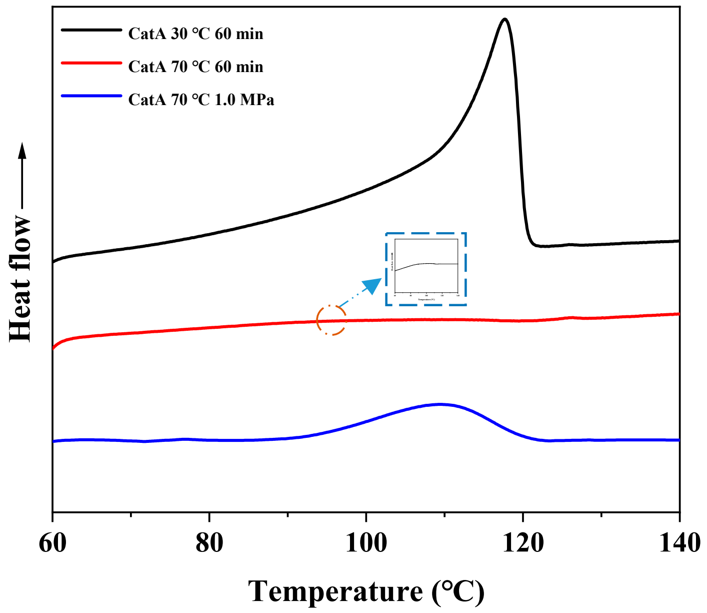

FigureS7 DSC curves of polyethylenes prepared by **CatA** in n-hexane solvent at different conditions (Entries 4, 8, 9 in Table 1)

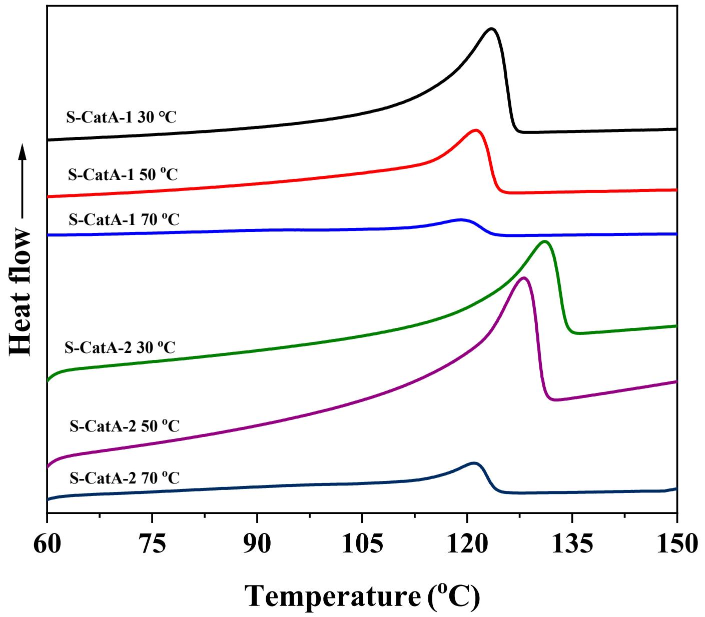

FigureS8 DSC curves of polyethylenes prepared with heterogeneous catalysts at different temperatures (Entries 1, 2, 3, 5, 6, 7 in Table 3)

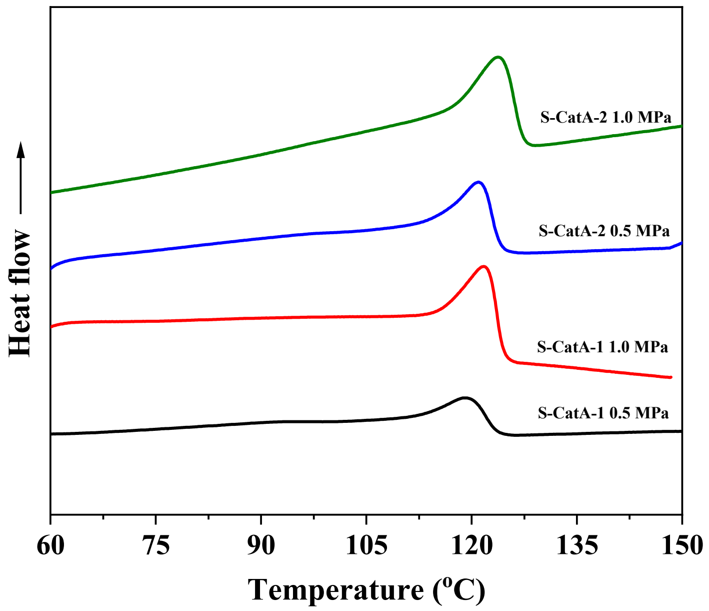

FigureS9 DSC curves of polyethylenes prepared **S-CatA-1/2** at different pressures at 70 °C (Entries 3, 4, 7, 9 in Table 3)

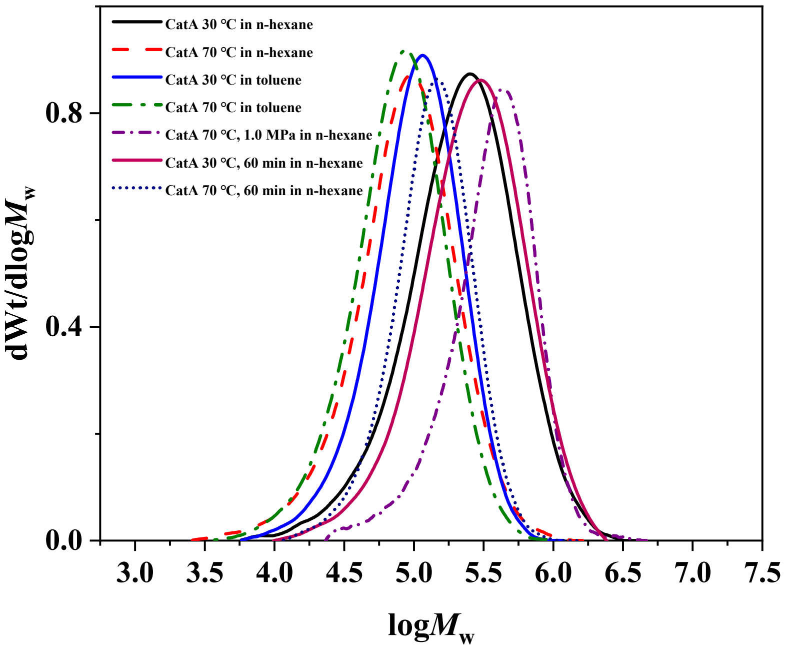

FigureS10 GPC curves of polyethylenes prepared by **CatA** at different conditions (Entries 1, 3, 4, 5, 7, 8, 10 in Table 1)

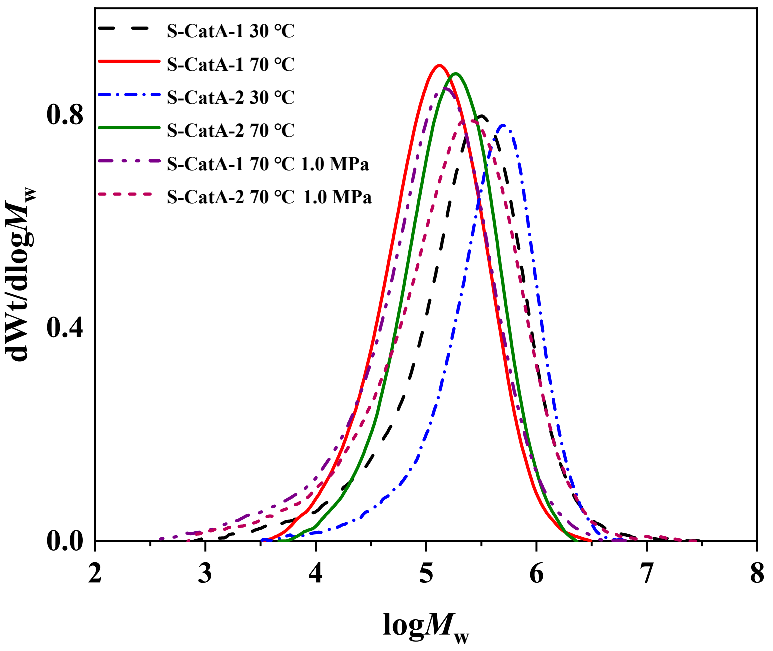

FigureS11 GPC curves of polyethylenes prepared by **S-CatA-1/2** catalysts (Entries 1, 3, 4, 5, 7, 9 in Table 3)

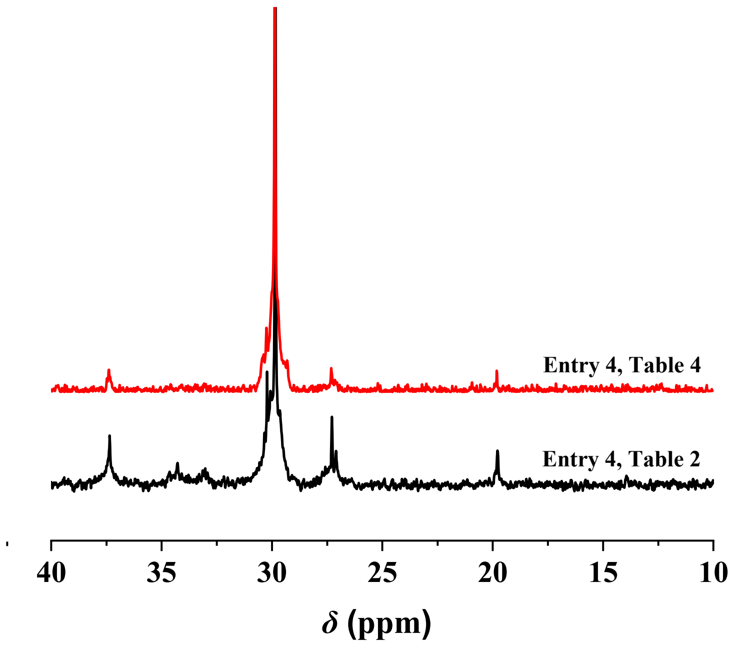

FigureS12 ^{13}C NMR spectra of polyethylenes prepared by **CatA**、**S-CatA-1** at 70 °C, 1.0 MPa (Entry 4 in Table 1, Entry 4 in Table 3)
